# PET/CT-based adaptive radiotherapy of locally advanced non-small cell lung cancer in multicenter yDEGRO ARO 2017-01 cohort study

**DOI:** 10.1186/s13014-022-01997-5

**Published:** 2022-02-09

**Authors:** Matthias Mäurer, Lukas Käsmann, Daniel F. Fleischmann, Michael Oertel, Danny Jazmati, Daniel Medenwald, Young DEGRO Trial Group

**Affiliations:** 1 Department of Radiation Oncology, University Medical Center Jena, Jena, Germany; 2 Department of Radiation Oncology, University Hospital, LMU Munich, Munich, Germany; 3 Department of Radiation Oncology, University Medical Center Muenster, Muenster, Germany; 4 Department of Particle Therapy, West German Proton Therapy Centre Essen (WPE), West German Cancer Center (WTZ), University Hospital Essen, Essen, Germany; 5 Department of Radiation Oncology, Faculty of Medicine, Martin Luther University Halle-Wittenberg, Halle (Saale), Germany

**Keywords:** Lung cancer, Non small cell lung cancer, PET/CT, Adaptive radiotherapy

## Abstract

**Background:**

Stage III non-small cell lung cancer (NSCLC) represents a highly heterogeneous disease and treatment burden. Advances in imaging modality show promising results for radiotherapy planning. In this multicentric study, we evaluated the impact of PET/CT-based radiotherapy planning on the prognosis of patients with stage III NSCLC.

**Method and patients:**

A retrospective observational cohort study (ARO 2017-01/NCT03055715) was conducted by the young DEGRO trial group of the German Society for Radiation Oncology (DEGRO) with the primary objective to assess the effect of tumour volume change during chemoradiotherapy and the secondary objective to assess the effect of treatment planning on survival. Three hundred forty-seven patients with stage III NSCLC treated at 21 university centers between January 2010 and December 2013 were enrolled in this trial. Patients received primary curative chemoradiotherapy with an intended dose of 50 Gy (hypofractionated) or > 60 Gy (normofractionated). To assess the effect of radiotherapy planning modality on overall survival, we used multivariate frailty models. Models were adjusted for gross tumor volume at the initiation of therapy, age, sex, simultaneous chemotherapy, lung comorbidities, RT dose and tumor grade. By considering the random effect, we can account for heterogeneity in survival and considered covariates within the model in relation to the study side.

**Results:**

Patients were predominantly male (n = 269, 78.4%) with mainly adenocarcinoma (56.4%) and an average of 67.2 years. Adaptation of radiotherapy with consecutive reduction of irradiation volume showed no significant disadvantage for patient survival (HR = 1.21, 95% CI 0.89–1.64). The use of PET/CT co-registration in radiation planning tended to result in better oncologic outcomes, although no significant association could be shown (HR = 0.8, 95% CI 0.56–1.16). Centers with a consistent planning strategy performed better than those without a preferred planning method (0.62, 95% CI 0.41–0.94).

**Conclusion:**

A consistent planning strategy has positive effects on overall survival. The use of PET/CT-based adaptive radiotherapy planning shows a similar survival prospect with the prospective of lower treatment volumes. In future research, toxicities need to be analysed in order to assess such reasoning.

## Introduction

Lung cancer is the leading cause of cancer-related deaths worldwide [1, 2]. The prognosis for patients with locally advanced stage III non-small cell lung cancer (NSCLC) remains poor despite the use of modern immunotherapies [3–5].

One strategy to improve the prognosis is an optimization of tolerability of treatment by more precise irradiation methods [6, 7]. Technological improvements in recent years have enabled dose escalation with better tumor coverage and optimized sparing of normal tissues, resulting in a survival advantage with lower toxicity [8–10]. These techniques include intensity-modulated radiotherapy, adaptive image-guided radiotherapy and the use of ^18^F-FDG PET/CT in radiation planning [11, 12]. In particular, information on metabolism provided by ^18^F-FDG PET/CT can improve target volume definition and dose planning before and during radiotherapy (RT), enabling better selection of patients and individualization of therapeutic strategies [13, 14]. A systematic review and meta-analysis could demonstrate a relevant change in target volume definition in about 40% of NSCLC with the use of a planning PET/CT [15]. Accordingly, the multicentric PET-plan study showed a possible isotoxic dose-escalation for the use PET-guided RT [10]. The use of further PET-tracers like FMISO may additionally tailor individual (and possibly smaller) metabolic tumor volumes [16]. However, the reduction of target volume for dose escalation has to be carefully balanced with adequate coverage of the tumor. In the RTOG 0617 trial, high-dose chemo-RT with 74 Gy did result in adverse overall survival (OS) when compared with standard dose RT 60 Gy, the later RT strategy revealing better dose coverage of the target [17, 18].

Despite the new data on the treatment of NSCLC, it is unclear how the radiation planning strategy and the use of PET/CT for radiation planning affect local control and survival. In addition, it is not known when and how often PET/CT should be performed in the setting of chemoradiotherapy and whether it should be performed as staging PET/CT or in the RT planning position. In the present analysis, we evaluated the relevance of ^18^F-FDG-PET-based radiotherapy planning on the prognosis of patients with stage III NSCLC in multicenter study including 347 patients.

## Methods and patients

### Study population, treatment and participating institutions

This retrospective observational cohort study (ARO 2017-01/NCT03055715) was conducted by the young DEGRO trial group (yDEGRO) of the German Society for Radiation Oncology (DEGRO). Twenty-one university centers for Radiation Oncology in Germany (n = 17), Spain (n = 1), Switzerland (n = 1), Belgium (n = 1) and Austria (n = 1) participated in the trial. Data of n = 347 patients who received curative-intent radiation therapy with curative intent (± chemotherapy) between January 1st 2010 and December 31st 2013 were analyzed.

Inclusion criteria were (1) inoperable UICC stage III A or B NSCLC (adenocarcinoma or squamous cell carcinoma) confirmed by biopsy, (2) CT-based radiation treatment planning (PET- or PET/CT-based if available), (3) completed curatively intended radiotherapy ± chemotherapy (planned total dose ≥ 60 Gy conventionally fractionated or ≥ 50 Gy hypo-fractionated) and (4) age ≥ 18 years. Patients with a secondary malignancy within 5 years prior to the diagnosis of the NSCLC and patients who received stereotactic body radiotherapy were excluded from the study.

Demographical, treatment, and clinical data was extracted from the patients’ clinical records at the participating sites and was collected using electronic case report forms (eCRF) which were stored in the RadPlanBio data base of the German Cancer Consortium (DKTK) and the German Cancer Research Center (DKFZ) [19]. Written informed consent of all patients was available prior to data acquisition and analysis.

### Statistics

To assess the effect of PET planning on overall survival we used multivariate frailty models. Models were adjusted for gross tumor volume (GTV) at the initiation of therapy, age, sex, simultaneous chemotherapy, lung comorbidities, RT dose and tumour grade. By considering the random effect, we can account for heterogeneity in survival and considered covariates within the model in relation to study side.

In another Cox regression models, we used ‘study center’ as predictor by forming four groups according to which method (no PET planning, PET co-registration, PET without co-registration) was the predominant choice of the respective center (≥ 50% of all cases planned by a one method). Center with no preferred method (no single planning method exceeding 50%) were merged to the fourth group.

In the models, we computed hazard ratios (HRs) with respective 95% confidence intervals (95%-CIs).

All analyses were performed with SAS, version 9.4.

## Results

The study included equal numbers of stage IIIA and IIIB patients (Table 1).

**Table 1:** Sociodemographic patient and disease characteristics

	Patient number (%)
Sex	
Male	269 (78.4%)
Female	74 (21.6%)
Age	Mean (SD): 67.2 (10.7)
Pack years	Mean (SD): 38.2 (25.1)
UICC stage (7th edition)	
IIIA	174 (50.1%)
IIIB	173 (49.9%)
Histology^a^	
Adenocarcinoma	134 (39.2%)
Squamous cell carcinoma	193 (56.4%)
Grading	
1	7 (2.0%)
2	104 (30.0%)
3	120 (34.6%)
4	5 (1.4%)
na^b^	111 (32.0%)
T stage	
T1	32 (9.2%)
T2	63 (18.2%)
T3	106 (30.5%)
T4	144 (41.5%)
TX	2 (0.6%)
N stage	
N0	32 (9.2%)
N1	42 (12.1%)
N2	172 (49.7%)
N3	97 (28%)
NX	3 (0.9%)
Sequential chemotherapy	
Yes	96 (28.0%)
No	247 (72.0%)
Dose	Mean (SD):
	63.5 (5.4)

^a^Adeno carcinoma, squamous-cell carcinoma 2 well, moderate, poor, undifferentiated (1–4)

^b^Not available

RT was combined with concurrent chemotherapy (CHT) in 250 patients (72.2%), 96 patients (27.8%) received sequential chemoradiotherapy. 75 patients (30%) were treated with combined cisplatin-vinorelbine CHT, 48 (19.2%) carboplatin-vinorelbine, 52 (20.8%) carboplatin-docetaxel, and 75 (30%) other chemotherapy doublet combinations.

In 314 (90.8%) patients’ conventional fractionation was used, compared to 7 (2%) patients were treated with hyperfractionated regimens, and 5 patients (1.5%) undergoing a simultaneous-integrated boost (SIB) concept. 20 patients (5.7%) received other RT concepts.

**Fig. 1:**
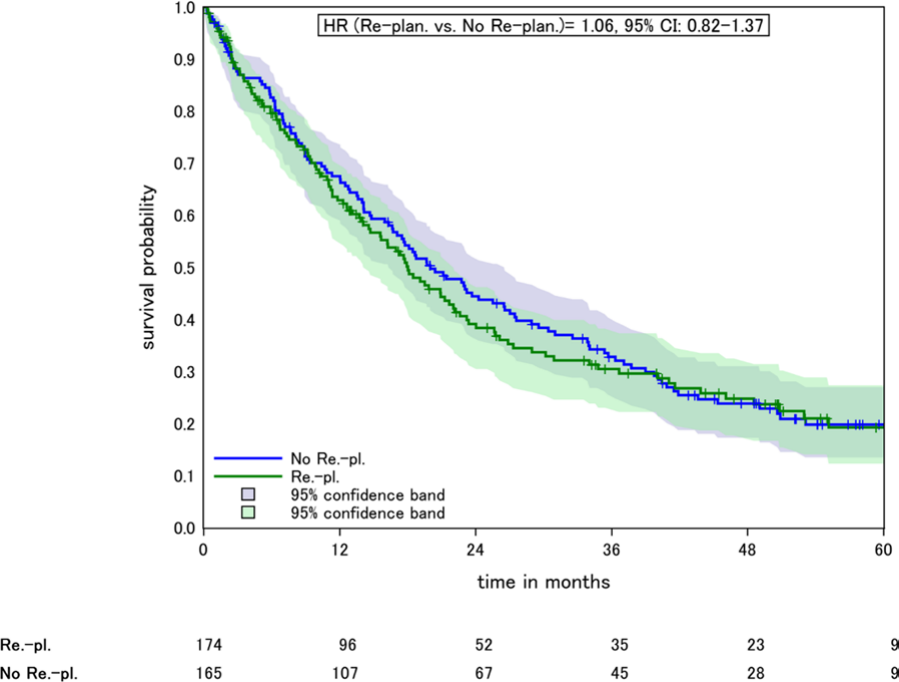
Kaplan–Meier plot of patients with (blue) and without (green) adaptive planning

In analysis of adaptive planning, no significant effect on survival was found for replanned cases when compared to cases with no re-planning (HR = 1.21, 95% CI 0.89–1.64, after covariate adjustment, Fig. 1, Table 1) (Table 2).

**Table 2:** Hazard ratios from frailty survival models using center as a random variable

	Crude^a^	Adj.^b^
Effect of re-planning		
No Re.-pl	Ref	Ref
Re.-pl	1.15 (0.84–1.57)	1.21 (0.89–1.64)
Effect of PET		
No PET	Ref	Ref
PET	0.85 (0.58–1.24)	0.91 (0.62–1.34)
PET coreg	0.76 (0.53–1.09)	0.8 (0.56–1.16)
Effect of PET usage in study centers		
PET coreg. versus No	0.83 (0.55–1.24)	0.72 (0.48–1.08)
PET coreg. versus PET	0.96 (0.63–1.46)	0.8 (0.54–1.19)
PET coreg. versus div	0.59 (0.37–0.94)	0.62 (0.41–0.94)

Adjusted for GTV1, age, sex, sim. Chemotherapy, lung comorbidities, RT dose, grade

^a^“center” as random variable, *Re.-pl.* re-planning, *coreg.* coregistration, *div.* diverse planning strategies

^b^Adj. for GTV1, age, sex, sim. Chemotherapy, lung comorbidities, RT dose, grade

**Fig. 2:**
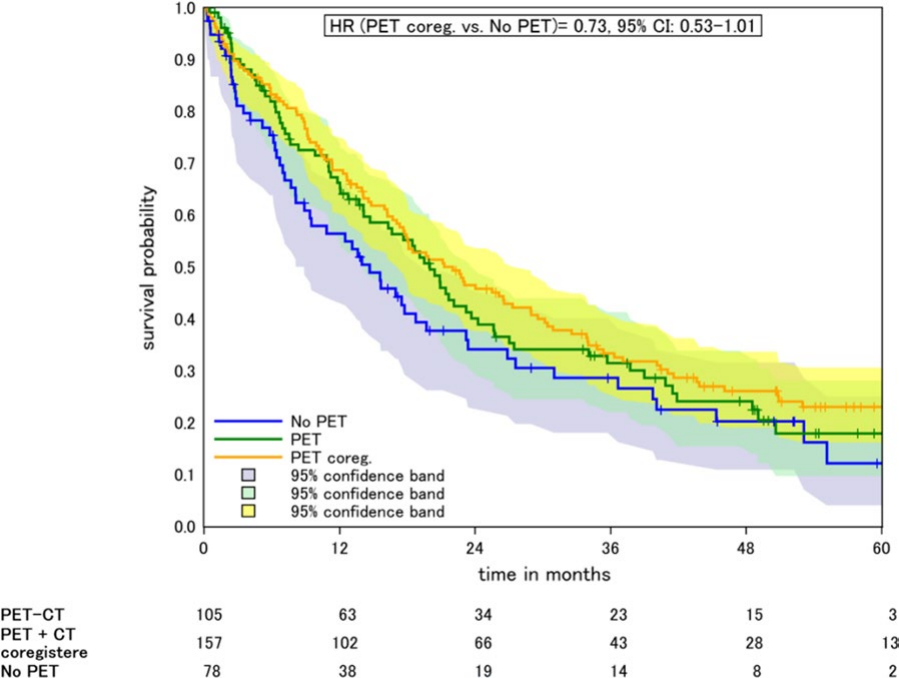
Kaplan–Meier plot of PET application

In the analysis of PET, cases with PET co-registration showed a similar survival rate as compared to cases without consideration of PET imaging (HR = 0.8, 95% CI 0.56–1.16 after covariate adjustment, Fig. 2, Table 1).

**Fig. 3:**
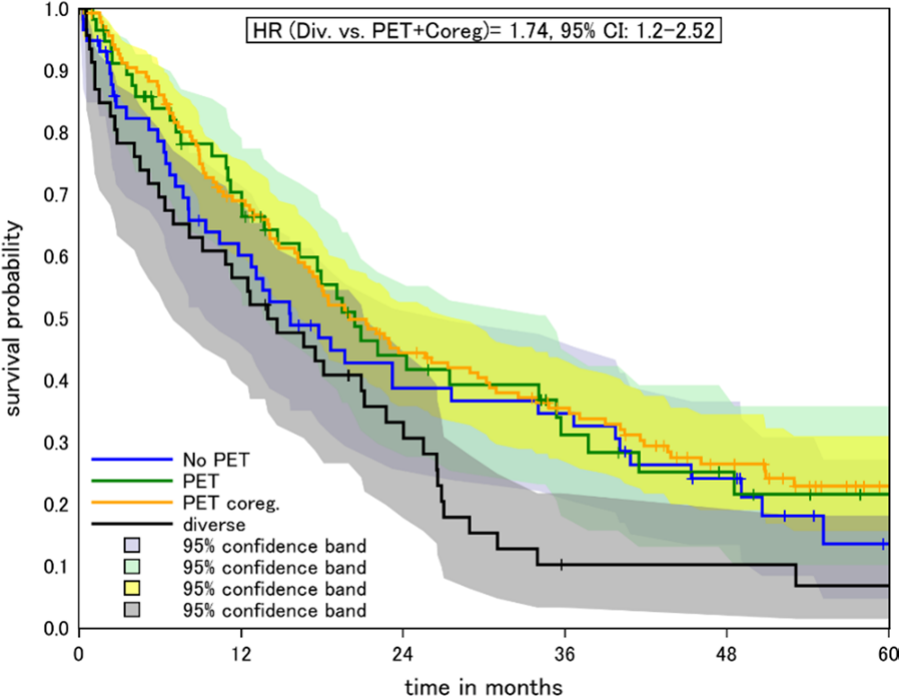
Kaplan–Meier plot of centers according to applied planning strategy

Analyzing centers according to the preferred planning strategy, we found that centers with no preferred method performed worse than those with a predominant planning method (0.62, 95% CI 0.41–0.94, after covariate adjustment, Fig. 3, Table 1). However, this finding is based on only one center in the mixed method group.

## Discussion

The present analysis demonstrates prognostic superiority of a consistent imaging strategy for advanced NSCLC. Our results confirm non-inferiority of target volume reduction in terms of outcome and therefore encourage PET/CT based RT planning for NSCLC.

Our results go along with the findings of Nestle et al. which could show that PET/CT-based reduction of radiotherapy target volume may improve local control without increasing toxicity in patients with locally advanced NSCLC [11]. The correct identification of PET-avid tumor tissue is pivotal as it acts as a starting point for local recurrence: a patterns-of-failure study on NSCLC patients demonstrates local recurrences to occur predominantly within the previous active volume [20].

The multicentric randomized PET-plan study demonstrates the ability of an isotoxic RT dose escalation (mean dose 65.3 Gy vs. 67.3 Gy for the standard vs. the experimental arm) with the use of 18F-FDG PET/CT for planning [10]. Despite smaller target volumes in the PET-arm, locoregional failure was not inferior (30% vs. 17% in the intention-to-treat population after 1 year for the standard vs. experimental arm) [10]. Consistent with our data, no significant impact on OS could be shown. However, a safe RT-volume reduction with improved sparing of healthy lung is likely to results in lower toxicity [21–23].

As shown previously by our group, we found a mean reduction in GTV volume at the time of re-planning of 48.2 mL or 31.1% [24].

Based on our findings, the consistency of centers in performing each standard, regardless of modality, appears to have a prognostic impact. Patients from centers with no stringently applied RT planning procedure experienced a worse outcome compared to centers with consistent RT planning protocols (CT versus PET/CT). In this respect, it seems advisable not to switch too frequently between different adaptive procedures, but to apply a homogenous in-house protocol.

Although not statistically significant patients with a co-registered PET actually numerically outperformed those with staging PET/CT. This is of importance as planning PET/CT are not mandatory in most studies: in RTOG 0617 around 90% of patients had a PET-staging in each arm, whereas its use for RT-planning was only encouraged [16].

Thus, the use of PET/CT in radiation planning (co-registered or in RT treatment position) should be considered in accordance with modern guidelines (ESTRO-ACROP NSCLC). From a public health perspective the application of PET-based RT planning was shown to be cost-effective when compared to CT-based planning [25]. Other concepts such as simultaneous integrated boost need to take account for altering treatment volumes [26]. As variability in the application of planning techniques might be associated with an adverse survival prospect PET-based planning might additionally contribute to a reduced heterogeneity in the definition of target structures [27].

At the time of enrollment to our study, sequential durvalumab maintenance implemented by the PACIFIC trial was not the standard of care for patients with inoperable stage III NSCLC, but needs to be taken into account today [23]. Importantly, PET/CT staging and treatment planning was not mandatory in the PACIFIC trial but should be considered standard of care based on our findings consistent with recent literature [11, 28]. Since increased lung toxicity has been previously reported in patients treated for stage III NSCLC with durvalumab, reduction of irradiated lung is of increasing importance [29].

From the results of our retrospective study, further direction of future research on RT treatment of patients with locally advanced NSCLC should focus on the possibilities of PET/CT-based RT planning regarding further improvement of local control monitored by PET/CT-based recurrence pattern analyses.

### Limitations

In our study the majority of institutions preferred one approach above others with only one centre using multiple planning strategies. In order to gain a broader perspective, the inclusion of more centers with different planning methods should be envisaged. Furthermore, as real-life data were used, there was no standardized method for target volume delineation. Thus, PET information might have been used differently for target volume delineation among study centres. Finally, PET imaging might lead to differences in clinical stages, especially in the amount of lymph node involvement. This might lead to an up- or down-staging of respective cases. However, in our study this might affect our results only in so far as the covariate adjusted models or inclusion criteria are concerned.

## Conclusion

A consistent radiotherapy planning strategy should be followed for patients undergoing definitive chemoradiotherapy for stage III NSCLC. The use of PET/CT-based adaptive radiotherapy planning shows comparable oncologic outcomes and should be considered to avoid radiogenic toxicities.

## Data Availability

The datasets generated during and/or analysed during the current study are not publicly available but may become available upon request with permission from corresponding author and host institution following sufficient maturation of data.

## Abbreviations

^18^F-FDG PET: ^18^F-fludeoxyglucose positron emission tomography
CT: Computed tomography
DEGRO: German Society for Radiation Oncology
DKFZ: German Cancer Research Center
DKTK: German Cancer Consortium
NSCLC: Non-small cell lung cancer
RT: Radiotherapy
UICC: Union for International Cancer Control
